# Contrasting patterns of nucleotide polymorphism suggest different selective regimes within different parts of the *PgiC1* gene in *Festuca ovina* L.

**DOI:** 10.1186/s41065-017-0032-6

**Published:** 2017-05-18

**Authors:** Yuan Li, Bengt Hansson, Lena Ghatnekar, Honor C. Prentice

**Affiliations:** Department of Biology, Lund University, Lund, Sweden

**Keywords:** *Festuca ovina*, Cytosolic phosphoglucose isomerase, *PgiC1*, Nucleotide polymorphism, Purifying selection

## Abstract

**Background:**

Phosphoglucose isomerase (PGI, EC 5.3.1.9) is an essential metabolic enzyme in all eukaryotes. An earlier study of the *PgiC1* gene, which encodes cytosolic PGI in the grass *Festuca ovina* L., revealed a marked difference in the levels of nucleotide polymorphism between the 5’ and 3’ portions of the gene.

**Methods:**

In the present study, we characterized the sequence polymorphism in *F. ovina PgiC1* in more detail and examined possible explanations for the non-uniform pattern of nucleotide polymorphism across the gene.

**Results:**

Our study confirms that the two portions of the *PgiC1* gene show substantially different levels of DNA polymorphism and also suggests that the peptide encoded by the 3’ portion of *PgiC1* is functionally and structurally more important than that encoded by the 5’ portion. Although there was some evidence of purifying selection (*d*_N_/*d*_S_ test) on the 5’ portion of the gene, the signature of purifying selection was considerably stronger on the 3’ portion of the gene (*d*_N_/*d*_S_ and McDonald–Kreitman tests). Several tests support the action of balancing selection within the 5’ portion of the gene. Wall’s *B* and *Q* tests were significant only for the 5’ portion of the gene. There were also marked peaks of nucleotide diversity, Tajima’s *D* and the *d*_N_/*d*_S_ ratio at or around a *PgiC1* codon site (within the 5’ portion of the gene) that a previous study had suggested was subject to positive diversifying selection.

**Conclusions:**

Our results suggest that the two portions of the gene have been subject to different selective regimes. Purifying selection appears to have been the main force contributing to the relatively low level of polymorphism within the 3’ portion of the sequence. In contrast, it is possible that balancing selection has contributed to the maintenance of the polymorphism within the 5’ portion of the gene.

**Electronic supplementary material:**

The online version of this article (doi:10.1186/s41065-017-0032-6) contains supplementary material, which is available to authorized users.

## Background

Levels of nucleotide polymorphism have been shown to vary greatly between different parts of the genome (e.g. [1–3]), and there may also be variation in the levels of polymorphism within individual genes (e.g. [4–7]). A non-uniform pattern of nucleotide polymorphism within genes may arise if different types of selective pressure are operating on different regions of the gene (cf. [8, 9]). Different regions of a gene may code for peptides that have different structural or functional significances, and the regions of a gene with more stringent structural and/or functional requirements are expected to be subject to stronger purifying selection [10] and, therefore, tend to show lower levels of nucleotide polymorphism than regions that are subject to less stringent constraints [11, 12]. Positive directional selection may reduce the levels of local nucleotide polymorphism within a gene [9], while balancing selection may increase the levels of nucleotide polymorphism at, and in the vicinity of, the selected sites [13, 14]. A classic example of a case where selection results in non-uniform levels of nucleotide polymorphism between different gene regions is that of the major histocompatibility complex (MHC) genes. These genes are crucial for the ability of a vertebrate host’s immune system to detect evolving pathogens, and it is frequently suggested that the maintenance of the high levels of non-synonymous polymorphism in the MHC gene regions encoding the antigen binding site is a reflection of pathogen-driven balancing selection [15, 16]. In addition to selective processes, varying rates of recombination and mutation, as well as stochastic processes, may also contribute to non-uniform levels of nucleotide polymorphism between different regions of a gene (cf. [11, 17]).

**Fig. 1:**
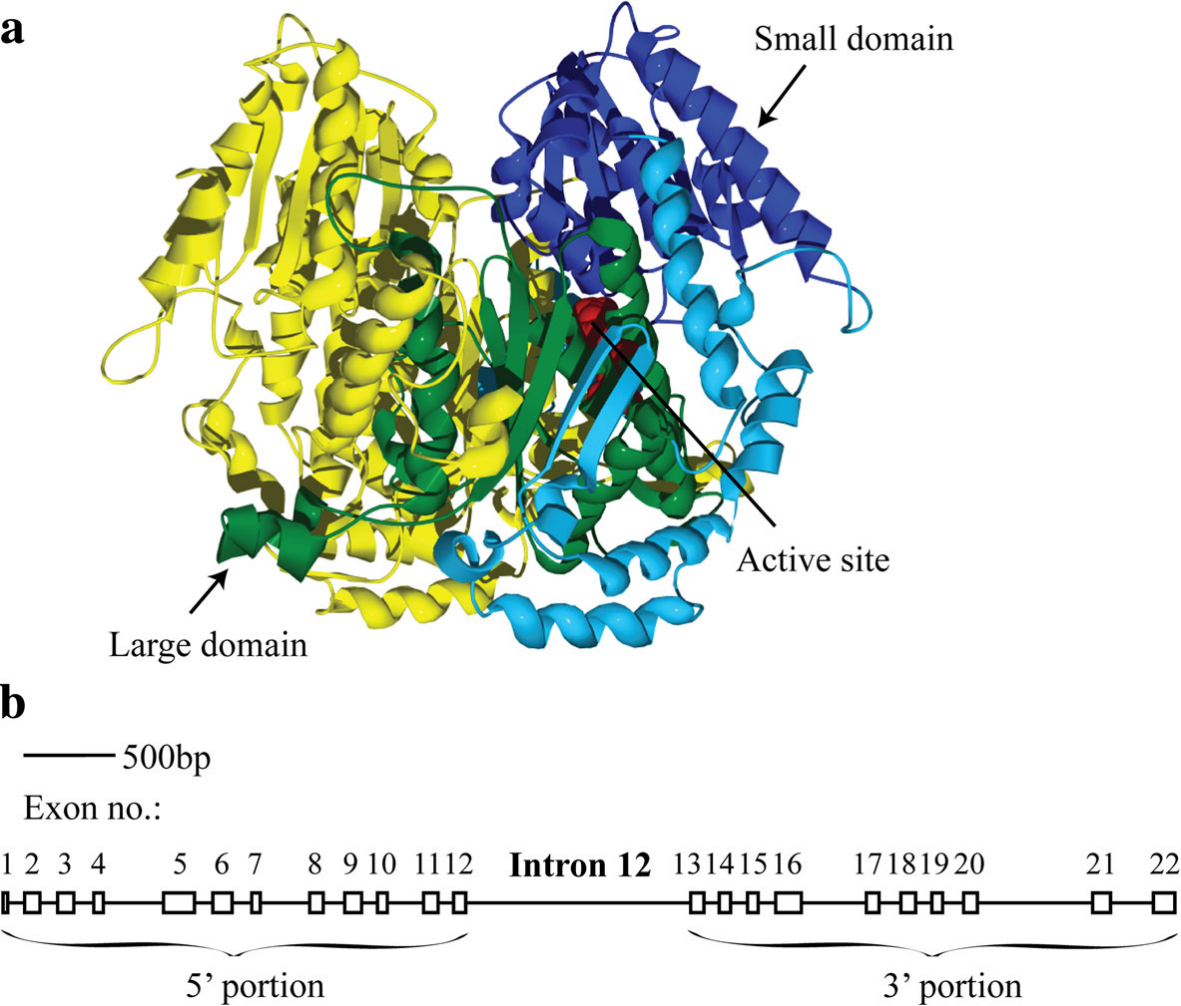
The structure of the *PgiC1* gene and the 3-D protein structure of its gene product. **a** PGI dimer, coded for by *PgiC1*, in *F. ovina* and homology modelled in an earlier study [34]. One monomer is shown in *yellow*. In the other monomer, the large domain is shown in *green* and the small domain is shown in *dark blue*. The three active site residues (equivalent to Lys516, Glu360, and His391 in *F. ovina*) that are directly involved in the PGI isomerization reaction are shown in *red*. The rest of the monomer is represented in *light blue*. **b** The gene structure is summarized for the part of the *PgiC1* gene corresponding to the 1 633 bp sequence of the 29 Öland sequences characterized in the present study. *Boxes* represent the exons and lines represent introns. The gene structure is scaled according to an earlier published *PgiC1* gene sequence (GenBank accession numbers HQ616103). The 5’ and 3’ portions (see Fig. 2) of the *PgiC1* gene that are compared in the present study include, respectively, exons 1–12 and exons 13–22

The *PgiC1* gene, which encodes the cytosolic version of the metabolic enzyme phosphoglucose isomerase (PGI, EC 5.3.1.9), in the grass *Festuca ovina* L., represents one of the few reported cases in which the levels of nucleotide polymorphism differ substantially between the 3’ and 5’ portions of a gene [18]. PGI catalyses the second step of glycolysis [19], and is also known to have diverse moonlighting functions (see the references in [20]). The functional PGI enzyme is formed by two monomers, with each monomer being composed of two main domains (the “small domain” and the “large domain”) [21, 22] (Fig. 1-a). High levels of allozyme/isozyme variation have been frequently reported for PGI in many different species [23]. Observed differences in enzyme activity between PGI variants in a number of species are consistent with observed associations between the PGI variation and environmental variables or life-history traits – suggesting that the loci coding for PGI may be under selection (e.g. [24, 25]).

*Festuca ovina* is a perennial, tussock-forming and outcrossing grass, with wind-dispersed pollen and seeds [26]. The species has a broad ecological amplitude and is widespread in unfertilized grasslands in northern Europe (e.g. [26, 27]). The steppe-like “alvar” grasslands on the Baltic island of Öland (Sweden) are characterized by a fine-scale edaphic mosaic, with moist and dry, and high and low pH microhabitats. Earlier studies suggest that cytosolic PGI isozyme variation in *F. ovina* may be involved in fine-scale microhabitat adaptation on Öland [26, 28, 29]. Analysis of replicated samples from different alvar sites shows that, despite the fact that *F. ovina* is strongly outcrossing, the frequencies of different cytosolic PGI isozyme electromorphs are significantly associated with microhabitat variation in the alvar grasslands and that electromorph frequencies change in response to experimental habitat manipulation [26, 28].

In *F. ovina*, cytosolic PGI is coded for by two loci, *PgiC1* and *PgiC2* [30]. The “native” *PgiC1* locus is present in all *F. ovina* individuals, whereas *PgiC2* is only present in some individuals and appears to have been horizontally acquired from a distantly related grass genus [29, 31–33]. Earlier analyses of the *PgiC1* gene in *F. ovina* suggest that two *PgiC1* amino acid codon sites may be affected by positive selection [34], and SNP (single nucleotide polymorphism) alleles at these two codon sites show significant associations with microhabitat variables in the alvar grasslands (Y Li, B Hansson, M Lönn, HC Prentice, unpublished results).

The uneven distribution of polymorphic nucleotide sites along the *PgiC1* gene was noted in an earlier study that included five *PgiC1* coding sequences from Skåne, S Sweden [18]. The longest intron (intron 12, Fig. 1-b) was used as a demarcation point between the polymorphic 5’ portion of the gene and the, substantially less polymorphic, 3’ portion of the gene. The aim of the present study was to investigate the possible evolutionary mechanisms that may have contributed to the contrasting levels of nucleotide polymorphism in the two portions of the *PgiC1* gene in *F. ovina*. We analysed the levels of *PgiC1* nucleotide polymorphism within a larger dataset (29 *PgiC1* cDNA sequences) from *F. ovina* individuals collected from the alvar grasslands on Öland, and carried out a range of tests to assess the relative importance of different types of selection that may have contributed to the non-uniform pattern of nucleotide polymorphism within *PgiC1*.

**Fig. 2:**
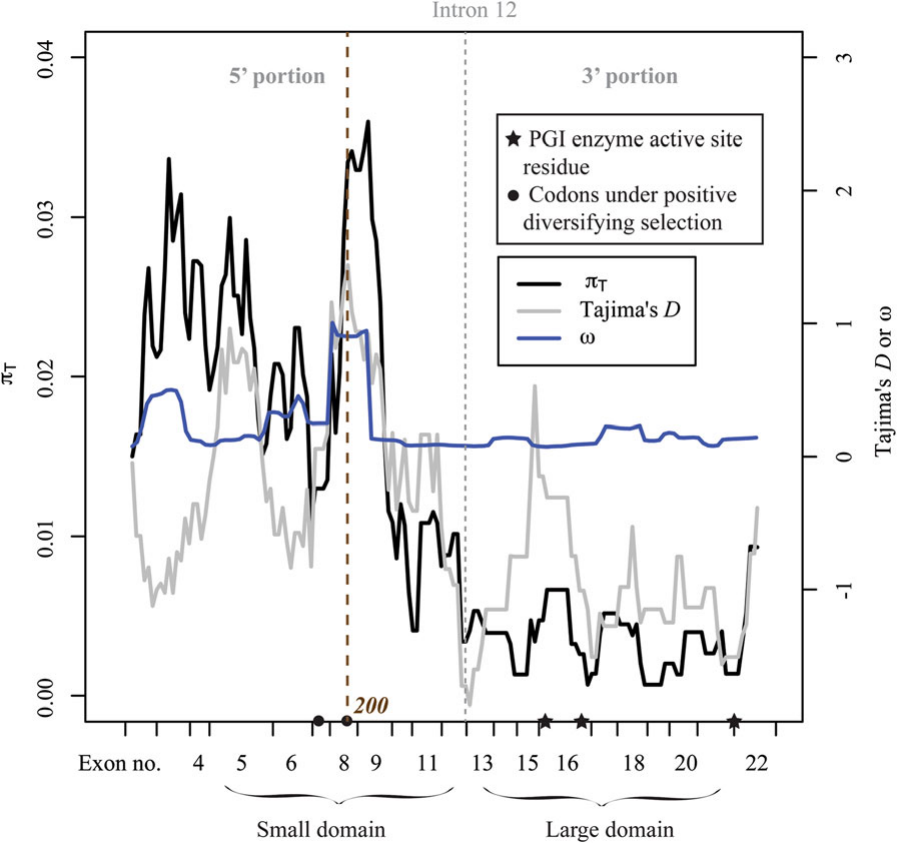
Sliding window analyses of nucleotide diversity (*π*_T_), Tajima’s *D* and *ω*. The ticks on the x axis represent the boundary of each analysed *PgiC1* exon within the *PgiC1* coding sequence. In *F. ovina*, *PgiC1* exons 5–12 encode the small domain of a PGI monomer while exons 13–21 encode the large domain. The two dots on the *x axis* show the locations of the two *PgiC1* codon sites (173 and 200) that were earlier identified as being candidates for positive diversifying selection [34]. The three stars on the *x axis* represent the three active site residues (equivalent to Lys516, Glu360, and His391 in *F. ovina*) that are directly involved in the PGI isomerization reaction [35]. The *grey dotted vertical line* shows the location of intron 12, which is used as the demarcation point for defining the 5’ and 3’ portions of *PgiC1* sequence. The *brown dotted vertical line* indicates codon site 200 which is under positive diversifying selection and located at or near to peaks of *π*_T_, Tajima’s *D* and *ω*

The 3’ portion of *PgiC1* in *F. ovina* encodes the structurally important large domain and three functionally essential active site residues (Figs. 1-a and 2). The extensive inter-monomer interaction between the large domains of the two monomers is necessary for the formation of a stable PGI dimer [21] and the three active site residues (equivalent to Glu360, His391 and Lys516 in *F. ovina*) participate directly in the isomerization reaction of PGI [35]. If the 3’ portion of *PgiC1* codes for products that are subject to greater structural or functional constraints than the products of the 5’ portion of the gene, then a relatively stronger level of purifying selection (i.e. negative selection) may be expected to have contributed to the low level of nucleotide polymorphism within the 3’ portion of *PgiC1* in *F. ovina*. The 5’ portion of *PgiC1* contains the amino acid codon sites 173 and 200 (Fig. 2). If these sites are under balancing selection (i.e. positive intraspecific diversifying selection), as suggested by [34], then balancing selection targeting the two sites might be expected to contribute to the high level of nucleotide polymorphism within the 5’ portion of *PgiC1*. The present study provides support for the prediction that there is a stronger purifying selection on the 3’ portion than on the 5’ portion of *PgiC1*, and suggests that there is balancing selection on the 5’ portion of the gene.

## Methods

### Plant material and sequences

The present study examined variation within 29 *PgiC1* cDNA sequences (GenBank accession numbers KF487737-KF487765, [34]) from Öland populations of *F. ovina*. The sequences were derived from 15 individuals that were chosen to represent the five cytosolic PGI electromorphs (EMs 1, 2, 4, 5 and 6) that occur most frequently within populations of *F. ovina* on Öland [26, 28, 29] – with a particular focus on the two most common electromorphs, EM 1 and EM 2 [34]. The sequences were obtained by, first, synthesizing the total cDNA from the total RNA of each studied *F. ovina* individual [34]. The *PgiC1* cDNA was then PCR-amplified from the synthesized total cDNA, and the amplified *PgiC1* cDNA was cloned and sequenced [34]. Two *PgiC1* cDNA alleles were acquired from each (diploid) individual, giving a total of 30 alleles from the 15 studied individuals [34]. However, one of the alleles (GenBank accession number KF487766) contained an aberrant (113 bp) insertion [34] and was excluded from the analyses in the present study, unless specified.

Each of the 29 analysed *PgiC1* cDNA sequences covers 96% (1 633 bp) of the full-length (1701 bp, excluding stop codon) *PgiC1* coding sequence, and ranges from exon 1 to exon 22 (Fig. 1-b). For comparative purposes, we also downloaded the five Skåne *F. ovina PgiC1* coding sequences (GenBank accession numbers DQ225731-DQ225735) which were examined in the earlier study that noted the difference in the levels of polymorphism between the 5’ and 3’ portions of *PgiC1* [18]. These five Skåne sequences represent the common cytosolic PGI isozyme electromorphs 1, 2 and 6, as well as the rare electromorph 8 [26, 28]. Each of the five sequences covers 1 182 bp, out of the full-length *PgiC1* coding sequence [32], and ranges from exons 5 to 11 and from exons 13 to 21.

### Analysis of sequence data

The number of polymorphic sites (*S*), nucleotide diversity (*π*; [36]), Watterson’s estimator of the population mutation rate (*θ*_W_; [37]), and the number of haplotypes (*N*_h_) were calculated using DnaSP v. 5.10.01 [38]. All the statistics were calculated, separately, for the two portions of the five Skåne *PgiC1* sequences (5’: exons 5-11; 3’: exons 13-21) and of the 29 Öland (5’: exons 1-12; 3’: exons 13-22) *PgiC1* sequences. Total nucleotide diversity (*π*_T_) was estimated separately for each of the 22 studied *PgiC1* exons for the 29 Öland *PgiC1* sequences. Sliding window analysis of *π*_T_ was also carried out for the 29 Öland *PgiC1* sequences using DnaSP v. 5.10.01 (window length: 100 bp; step size: 10 bp). The remaining analyses only considered the 29 Öland *PgiC1* sequences, unless specified.

The level of recombination was estimated (as the minimum number of recombination events, *R*_M_, using the method of Hudson RR and Kaplan NL [39] as implemented in DnaSP v. 5.10.01) for each of the two *PgiC1* gene portions in the 29 Öland sequences. The level of recombination was also estimated as the population recombination rate (*ρ* = 4*N*_e_ *r*, where *N*e is the effective population size and *r* is the per-generation per-site recombination rate [40]), using the program omegaMap [41]. We used the same procedure as in [34] to run omegaMap, but used a sliding window of 10 codons to estimate *ρ* in the present study. The level of linkage disequilibrium (LD) within *PgiC1* was estimated using *r* ^2^ statistics [42], calculated between all pairs of polymorphic sites (excluding a single site that segregates with more than two nucleotides [34]), using Haploview v. 4.2 [43]. The genotypes of the 15 studied Öland individuals [34], at each of the analyzed polymorphic sites, were used as input to the Haploview analyses. In order to generate a complete set of genotype data for the 15 individuals, as required for the Haploview analyses, we included the additional sequence (GenBank accession number KF487766) – acquired from the 15 *F. ovina* individuals but containing an aberrant insertion that may have resulted from incomplete splicing of the *PgiC1* precursor mRNA [34]. This insertion was removed for the Haploview analyses.

The *d*N/*d*S ratio (*ω*) (*dN* = non-synonymous substitution rate; *dS* = synonymous substitution rate) was estimated, together with *ρ* (using omegaMap), for each amino acid codon translated from the *PgiC1* sequence. The estimated *ω* value was used to examine whether purifying (i.e. negative selection, *ω* < 1) or balancing (i.e. positive intraspecific diversifying) selection (*ω* > 1) may have contributed to the amounts and patterning of sequence variation within and between the two gene portions for the 29 studied *PgiC1* sequences (cf. [41]). Sliding window analysis of *ω* was also carried out (manually, on the basis of the results from OmegaMap) with a window length of 99 and a step size of 12.

Neutrality tests, including the Hudson-Kreitman-Aguadé (HKA) test [11], Tajima’s *D* test [44], Fay and Wu’s *H* test [45], MacDonald and Kreitman’s (MK) test [46] and Wall’s *B* and *Q* tests [47] were also used to examine whether selection may have contributed to the amounts and patterning of sequence variation within each of the two *PgiC1* gene portions in the 29 sequences*.* All the neutrality tests were carried out using DnaSP v. 5.10.01. The significances of the Tajima’s *D*, Fay and Wu’s *H*, and Wall’s *B* and *Q* were conservatively estimated (without allowing for recombination), using 10 000 coalescent simulations and on the basis of the observed number of segregating sites. A single *PgiC* cDNA sequence from *F. altissima* (GenBank accession number DQ225740), encoding cytosolic PGI, was used as the outgroup for the HKA test, Fay and Wu’s *H* test and the MK test. We used the HKA test to compare the 5’- and 3’-portions of *PgiC1* in terms of intraspecific polymorphism (within *F. ovina*) and interspecific divergence (between *F. ovina* and *F. altissima*)*.* In the MK test, we compared the ratio of *D*_N_/*D*_S_ (*D*_N_ or *D*_S_ = the number of fixed non-synonymous [for *D*_N_] or synonymous [for *D*_S_] substitutions per gene between *F. ovina* and *F. altissima*), with the ratio of *P*_N_/*P*_S_ (*P*_N_ or *P*_S_ = the number of non-synonymous [for *P*_N_] or synonymous [for *P*_S_] polymorphic sites per gene within *F. ovin*a). The degree of synonymous divergence (*K*_S_ [JC]: Jukes-Cantor corrected number of synonymous substitutions per synonymous site) between the outgroup *PgiC* sequence and the 29 *F. ovina PgiC1* sequences was 0.240. Sliding window analyses were also carried out, respectively, for Tajima’s *D*, *ω*, Fay and Wu’s *H*, as well as for *K*a/*K*s (the number of nonsynonymous substitutions per nonsynonymous site/the number of sysnonymous substitutions per synonymous site), with a window length of 100 bp and a step size of 10 bp using DnaSP v. 5.10.01.

### Analysis of the evolutionary conservation of amino acid sites

The degree of evolutionary conservation at each of the respective PGI amino acid sites corresponding to the *PgiC1* translated amino acid sites was estimated, on the basis of the phylogenetic relationships among a large set of homologous sequences, from a wide range of different species, using the online application ConSurf Server [48]. The database UniRef90 [49] was searched for sequences that were homologous with the *F. ovina PgiC1* input sequence, using CSI-BLAST [50] (cutoff E-value = 0.0001; number of interactions = 3; maximum homologs to collect = 150). Within CSI-BLAST, redundant sequences were filtered out by clustering blast hits with a sequence identity of 95% or more and only using one representative of each cluster in the analysis. BLAST hits that shared a sequence identity of less than 35% with the input sequence were ignored. A multiple-species alignment (Additional file 1: File S1) of the acquired homologous sequences was constructed using MAFFT [51, 52], and this alignment was then used to build a phylogenetic tree using the neighbour-joining algorithm as implemented in the Rate4Site program [53]. The level of evolutionary conservation was then estimated, as a conservation score for each amino acid site using an empirical Bayesian algorithm [54] implemented in the ConSurf Server. The lower the conservation score, the more evolutionarily conserved are the amino acid residues at that specific site. The translated amino acid sequence from a *PgiC1* sequence (GenBank accession number KF487738), representing the most common *PgiC1* sequence in *F. ovina*, was used as the input to the ConSurf Server.

## Results

### Sequence variation

The analyses of the five Skåne *PgiC1* coding sequences confirmed the earlier finding [18] that the 5’ portion of the gene (*L* [sequence length] = 570, *S* = 30, *N*_h_ = 5, *π* = 0.025, *θ*_W_ = 0.025) was considerably more polymorphic than the 3’ portion (*L* = 612, *S* = 3, *N*_h_ = 4, *π* = 0.002, *θ*_W_ = 0.002). Also in agreement with the observed pattern of sequence polymorphism within the five Skåne *PgiC1* sequences, the analyses of the 29 Öland *PgiC1* cDNA sequences showed that the 5’ portion of the sequence (*π*_T_ = 0.019, *θ*_W_ = 0.020) was substantially more polymorphic than the 3’ portion of the sequence (*π*_T_ = 0.004, *θ*_W_ = 0.007) (Fig. 2, Table 1). There was a significant difference between the *π*_T_ values for each of the 12 exons (exons 1–12) within the 5’ portion of the *PgiC1* gene in the 29 sequences, and those for each of the 10 exons (exons 13–22) within the 3’ portion of the 29 sequences (Wilcoxon rank sum test; W = 96, *P* = 0.019, Additional file 2: Table S1). The sliding window analyses of *π*_T_ showed that the *PgiC1* codon site 200 (under positive diversifying selection [34]) was within the highest peak of *π*_T_ at exon 8 within the 5’ portion of the gene (Fig. 2).

**Table 1:** Analyses of the 3’ and 5’ portions of 29 *PgiC1* sequences from Öland *F. ovina*

	*L*	*S*	*N*_h_	*π* (×10^−3^)	*θ*_W_ (×10^−3^)	*R*_M_	*ρ*	Mean *ω*	Tajima’s *D*	Fay & Wu’s *H*	Wall’s *B*	Wall’s *Q*	Mean conservation score
5’ portion	872	68	21	19	20	20	0.383	0.280	−0.195 n.s.	3.355 n.s.	0.015*	0.030*	0.105
3’ portion	761	20	15	4	7	1	0.026	0.128	−1.363 n.s.	−1.768 n.s.	0.000 n.s.	0.000 n.s.	−0.121

The table shows the lengths of the 5’ and 3’ portions of the sequence (*L*), the number of segregating sites (*S*), the number of haplotypes (*N*_h_), the total nucleotide diversity (*π*), the total Watterson’s estimator of population mutation rate (per site) (*θ*_W_), the minimum number of recombination events (*R*_M_), the population recombination parameter (*ρ*) (per site), the average *ω* ratio, Tajima’s *D*, Fay & Wu’s *H*, and Wall’s *B* and *Q*, as well as the mean conservation score (the smaller the value, the more conserved) estimated using the online ConSurf Server. Because parts of the coding sequence are not available for the outgroup sequence from *F. altissima*, the 5’ and 3’ portions of *F. ovina PgiC1* that were considered in the Fay and Wu’s *H* test span, respectively, coding sequence nucleotide positions 259-828 & 919-1530

*0.01 < *P* < 0.05; n.s. non-significant

### Recombination and linkage disequilibrium

**Fig. 3:**
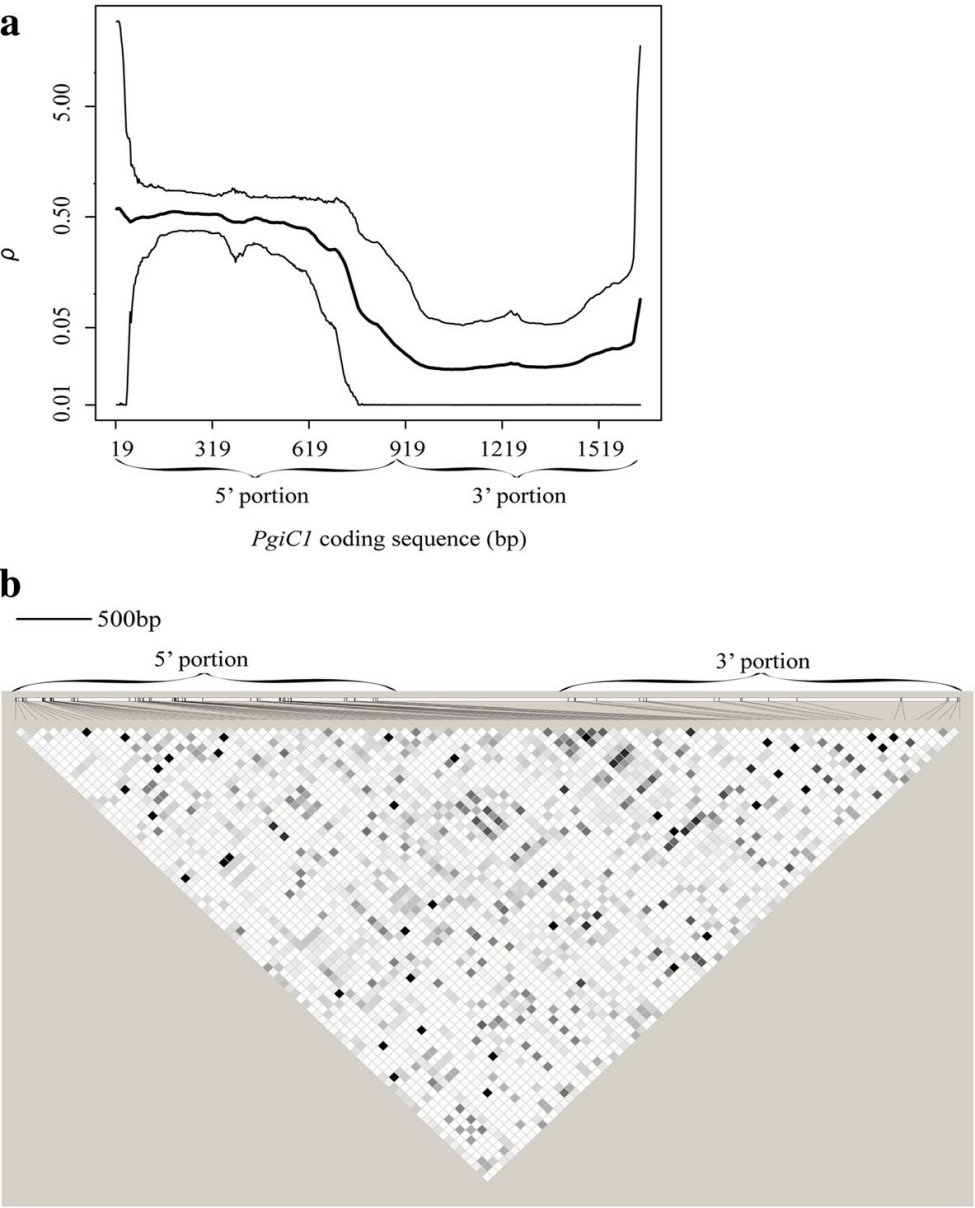
Levels of recombination and linkage disequilibrium (LD) within the *F. ovina PgiC1* sequence. **a** Population recombination rate (*ρ*) across *PgiC1*. The *heavy line* represents the mean value of *ρ*, while the *thin lines* represent the upper and lower 95% HPD (highest posterior density) interval bounds for the posterior distribution of *ρ*. **b** LD matrix for *PgiC1*. The level of LD is measured by the pairwise correlation coefficient *r* ^2^ values [42] for all the polymorphic nucleotide sites (except for one that segregates into more than two nucleotides [34]). Shades of *grey* indicate the *r* ^2^ values, ranging from *r* ^2^ = 0 (*white*) to *r* ^2^ = 1 (*black*). The proportional spacing of the polymorphic sites, which is scaled according to an earlier published *PgiC1* gene sequence (GenBank accession number HQ616103), is indicated by *black vertical lines* on a *white horizontal bar* (shown above the LD matrix)

The analyses of the 29 *PgiC1* sequences from Öland revealed a high overall level of recombination (*R*_M_ = 22; *ρ* = 0.217, Additional file 3: Table S2). The level of recombination in the highly polymorphic 5’ portion (*R*_M_ = 20; *ρ* = 0.383) was substantially higher than that for the less variable 3’ portion of the gene (*R*_M_ = 1; *ρ* = 0.026) (Table 1 and Fig. 3_a). However, the matrix of *r* ^2^ values (Fig. 3_b) shows that there is a low level of LD throughout the entire *PgiC1* gene, with no “strong LD” blocks (cf. [55]).

### The *ω* and neutrality tests

The fact that the overall *ω* (*d*N/*d*S ratio) value for the entire *PgiC1* sequence was substantially lower than 1 (mean *ω* over all the studied amino acid codons = 0.209, Additional file 3: Table S2) indicates that purifying selection has acted on the overall sequence. Purifying selection is also indicated for both the 5’ portion (291 amino acid codons; average *ω* = 0.280) and the 3’ portion (253 amino acid codons; average *ω* = 0.128) of the sequence. The fact that codons within the 3’ portion of the *PgiC1* sequence had a significantly lower *ω* than those in the 5’ portion (Wilcoxon rank sum test; W = 42229, *P* = 0.003) suggests that the 3’ portion may be under stronger purifying selection than the 5’ portion. The sliding window analyses of *ω* showed a marked plateau of *ω* values (between exons 8-9 within the 5’ portion) around the *PgiC1* codon site 200 (Fig. 2), that a previous study had suggested was subject to positive diversifying selection [34]. There were 40 segregating sites out of the total of 570 nucleotide sites included in the analysis of the 5’ portion, whereas there were only 14 segregating sites out of 612 sites in the 3’ portion (Additional file 4: Table S3). The levels of interspecific DNA divergence between the two gene portions were similar: 29.6 nucleotide differences among 570 sites for the 5’ portion, and 35.8 nucleotide differences among 612 sites for the 3’ portion (Additional file 4: Table S3). However the HKA test didn’t reject the null neutral model (*P* = 0.064, Additional file 4: Table S3).

Wall’s *B* and *Q* values were significant for the 5’ portion of the *PgiC1* sequence (Table 1), indicating an excess of LD between adjacent segregating sites, which may reflect balancing selection (cf. [47, 56]). Wall’s *B* and *Q* values were non-significant for the 3’portion of the *PgiC1* sequence (Table 1). There was a negative, but non-significant (*P* = 0.064, Table 1) Tajima’s *D* for the 3’ portion of the *PgiC1* sequence, while the Tajima’s *D* for the 5’ portion was near zero (*D* = -0.195, Table 1). The sliding window analyses of the Tajima’s *D* showed that the highest peak of *D* was located at the *PgiC1* codon site 200 (exon 8) (Fig. 2) which was identified as a potential target of positive diversifying selection in an earlier study [34]. The *D* value at this peak (*D* = 1.439) was near-significant (*P* = 0.060; post hoc significance test without correction for multiple tests). The Fay & Wu’s *H* values were non-significant for both the 5’ (*P* = 0.669) and the 3’ (*P* = 0.148) portions of the *PgiC1* sequence (Table 1). The sliding window analysis of Fay and Wu’s *H* showed that the deepest valley of *H* was at exon 16 (within the 3’ portion), near which a striking valley of Tajima’s *D* was also observed (Additional file 5: Figure S1). Both *D* and *H* had negative values in these valleys (Additional file 5: Figure S1), and according to the post hoc tests of significance (not subject to multiple test correction), the *H* was significant (*H* = -1.862, *P* = 0.006), while *D* was near-significant (*D* = -1.509, *P* = 0.095).

The MK test rejected the neutral null model for the 3’ portion of the *PgiC1* sequence (Fisher’s exact test, *P* = 0.018). The significant MK test reflects a lower *D*_N_/*D*_S_ ratio (0.064) relative to the *P*_N_/*P*_S_ ratio (0.556), which is consistent with purifying selection within this portion of the gene (Table 2). The MK test for the 5’ portion of the sequence was non-significant (*D*_N_/*D*_S_ = 0.235; *P*_N_/*P*_S_ = 0.323; Fisher’s exact test, *P* = 0.755, Table 2).

**Table 2:** MacDonald-Kreitman tests for the 3’ and 5’ portions of *F. ovina PgiC1*

Gene	Synonymous		Non-synonymous		Fisher’s exact test
Portion	Fixed^a^ (*D*_S_)	Poly^b^ (*P*_S_)	Fixed^a^ (*D*_N_)	Poly^b^ (*P*_N_)	*P-*value
5’ portion	17	31	4	10	0.755 n.s.
3’ portion	31	9	2	5	0.018*

Because parts of the coding sequence are not available for the outgroup sequence from *F. altissima*, the 5’ and 3’ portions of *F. ovina PgiC1* that were considered in the test span, respectively, coding sequence nucleotide positions 259-828 & 919-1530

^a^The number of fixed substitutions (per gene) between *F. ovina* and the outgroup (the number of nucleotide sites that are fixed for different nucleotides in *F. ovina* and the outgroup)

^b^The number of nucleotide polymorphic sites (per gene) within *F. ovina*

*0.01 < *P* < 0.05; n.s. non-significant

### Degree of amino acid site conservation

The PGI amino acid sites showed a tendency to be less evolutionarily conserved within the region corresponding to the 5’ portion of the translated *PgiC1* amino acid sequence (291 amino acid sites; mean conservation score = 0.105) than those corresponding to the 3’ portion of the translated *PgiC1* amino acid sequence (253 amino acid sites; mean conservation score = -0.121) (Wilcoxon rank sum test; W = 39886, *P* = 0.093) (Table 1, Additional file 6: Table S4).

## Discussion

Analyses of the 29 Öland *F. ovina PgiC1* cDNA sequences in the present study, together with the analyses of the five Skåne *PgiC1* sequences, shows that the nucleotide polymorphism is not evenly distributed within the *PgiC1* gene (Fig. 2). The 5’ portion of the *PgiC1* sequence is substantially more polymorphic than the 3’ portion, and our analyses suggest that the difference in the level of polymorphism may have resulted from different selective regimes in the two portions of the gene.

### Which evolutionary mechanisms may have contributed to the relatively low level of nucleotide polymorphism within the 3’ portion of the *PgiC1* sequence?

The parts of a protein that are more important for the stability and/or function of an enzyme are likely to be subject to stronger purifying selection [10] and, therefore, tend to exhibit a lower level of intraspecific polymorphism than the parts of the protein with less stringent functional and structural requirement [11, 12]. In *F. ovina*, the 3’ portion of the *PgiC1* sequence encodes a peptide that includes the structurally important large domain of the PGI monomer [21] and the three most conserved, functionally essential, active site residues [35]. The fact that the peptide translated from the 3’ portion of *PgiC1* contains important components of the 3-D structure of PGI suggests that this peptide may have a greater overall importance for the function of PGI than the peptide translated from the 5’ portion of *PgiC1*. The suggested difference in the functional and structural significance of the translated peptides between the two portions of *PgiC1* is supported by the estimated amino acid conservation scores in the present study, which show that the PGI amino acid sites corresponding to the 3’ portion of the *PgiC1* sequence tend to be evolutionarily more conserved than those of the 5’ portion in a wide range of species (cf. [48]).

If the peptide translated from the 3’ portion of *PgiC1* is more important for the function of the PGI enzyme than the peptide from the 5’ portion, then the 3’ portion of the gene may be expected to be under stronger purifying selection than the 5’ portion (cf. [10, 12]). In line with this expectation, the average *ω* value for the 3’ portion of *PgiC1* was considerably lower than that for the 5’ portion. The average values for both portions were much lower than 1 (suggesting purifying selection [41]). The fact that the value of Tajima’s *D* was more strongly negative for the 3’ portion (*D* = -1.363; *P* = 0.064) than for the 5’ portion (*D* = -0.195; *P* = 0.484) of the *PgiC1* sequence, is also consistent with the 3’ portion being under stronger purifying selection than the 5’ portion of the sequence (cf. [57]). The significant MK test result for the 3’ portion and the non-significance of the test for the 5’ portion of the *PgiC1* sequence may, again, suggest that the 3’ portion is under stronger purifying selection than the 5’ portion of the sequence. The significant MK test for the 3’ portion of *PgiC1*, with a lower ratio of fixed non-synonymous/synonymous substitutions between species (*D*_*N*_/*D*_*S*_ = 0.064) than the ratio of non-synonymous/synonymous polymorphism within species (*P*_*N*_/*P*_*S*_ = 0.556), suggests purifying selection, where the within-species nonsynonymous polymorphism that is maintained in selection–mutation balance consists mainly of weakly deleterious mutations [58].

Because the PGI protein structural elements are closely similar in a wide range of organisms (e.g. [21, 35, 59]), the functional and structural significance and the pattern of nucleotide polymorphism within the *Pgi* gene might be expected to be similar between *F. ovina* and other species. However, at present, only a few studies of *Pgi* have investigated gene-wide patterns of nucleotide polymorphism. Two of these studies, on the butterflies *Melitaea cinxia* [60] and *Colias eurytheme* [61, 62], reveal a nearly uniformly high level of nucleotide polymorphism across the entire *Pgi* gene. The pattern of nucleotide polymorphism in these two species was interpreted in terms of balancing selection (targeting a few amino acid sites located within the large domain) and moderate to high levels of LD within the entire *Pgi* gene [60, 61]. A high level of synonymous polymorphism was observed regionally around a nonsynonynous mutation within the 5’ portion of the *PgiC* gene in *Arabidopsis thaliana*, and interpreted in terms of balancing selection and an overall low level of recombination [63].

In contrast to the *Pgi* genes of the three species mentioned above, the *F. ovina PgiC1* gene showed a high level of recombination (*R*_M_ = 22): *M. cinxia* had a *R*_M_ of 6 [60], whereas *C. eurytheme R*_M_ = 11 [61] and *A. thaliana* showed no clear evidence of recombination [63]). The high estimated recombination value is consistent with the fact that *F. ovina* is a highly outcrossing species [26], and may indicate that different parts of the sequence could have evolved (at least to some extent) independently, resulting in a non-uniform pattern of nucleotide polymorphism across the sequence. Most of the identified recombination is within the 5’ portion of the *PgiC1* sequence, while the 3’ portion of the sequence shows limited recombination. However, the relatively low level of recombination detected for the 3’ portion of the sequence may be a consequence of purifying selection having removed variation at both the sites under selection and linked neutral sites (cf. [64]) – thereby removing the molecular signatures of recombination and lowering the numbers of identified recombination events (cf. [65]). The hypothesis that purifying selection may have removed the detectable signatures of recombination within the 3’ portion of *PgiC1* agrees with the *PgiC1* LD matrix, where a uniformly low level of LD is observed along the entire *PgiC1* cDNA sequence.

In addition to purifying selection, a selective sweep may also have contributed to the low level of nucleotide diversity within the 3’ portion of the gene: the near, but non-significant (*P* = 0.064), result of the HKA test suggests that variation-reducing selective forces may be acting on the *PgiC1* 3’ portion and/or variation-increasing forces acting on the 5’ portion (cf. [11]). The sliding window analyses revealed striking valleys of both Fay and Wu’s *H* and Tajima’s *D* at exon 16 (within the 3’ portion) (Additional file 5: Figure S1). In these valleys, the Fay and Wu’s *H* is significantly negative (*H* = -1.862; *P* = 0.006) and the Tajima’s *D* is also near-significantly negative (*D* = -1.509; *P* = 0.095). These negative values reflect a high-frequency of derived SNPs (around the valleys), suggesting a selective sweep (cf. [45]). A nearby shallow valley of total nucleotide diversity (Fig. 2) is also suggestive of a selective sweep [9]. The valleys of *D* and *H* at exon 16 are close to active site residue His391 (Additional file 5: Figure S1). Sequence patterns that are identified as the signatures of selection in neutrality tests (e.g. Tajima’s *D* and Fay and Wu’s *H*) may also result from factors such as population size changes or reflect population structure [66–68]. In the case of the highly outcrossing populations of *F. ovina* on Öland, population structure is unlikely to be a confounding factor in the neutrality tests. However, because possible confounding effects resulting from changes in population size cannot be excluded, the selective sweep suggested by the *H* and *D* tests should be interpreted with caution.

### Which evolutionary mechanisms may have contributed to the relatively high level of nucleotide polymorphism within the 5’ portion of the *PgiC1* sequence?

An earlier study [34] identified two *F. ovina PgiC1* codon sites (sites 173 and 200) as candidate targets of balancing selection (i.e. positive intraspecific diversifying selection) with a considerably stronger signal of selection for site 200 than for site 173 [34]. The sliding window analyses in the present study support the results of the earlier study and reveal a marked plateau of *ω* around the selected site 200 (but no peak associated with site 173) (Fig. 2). Protein structure modelling suggests that the translated amino acid polymorphism at these two *PgiC1* sites may affect either the interaction between the two monomers, or the domain-domain packing of the encoded PGI enzyme and, thus, influence the biochemical properties of the cytosolic PGI enzyme in *F. ovina* [34]. Biochemical studies in humans have shown that mutations at a few amino acid sites, which have similar 3-D structural locations to the two selected amino acid sites in *F. ovina*, significantly affect the activity of the PGI enzyme [69, 70].

Within *F. ovina PgiC1*, both codon sites 173 and 200, which were previously identified as candidates for balancing selection [34], are located within the 5’ portion of the sequence (Fig. 2). The significant results from the Wall’s *B* and *Q* tests (Table 1) support the suggestion that there has been balancing selection on the 5’ portion of the *PgiC1* sequence in *F. ovina* (cf. [47]). In addition to the significant *B* and *Q* tests, signals of balancing selection were also detected at the putative selected site 200. The highest peaks of, respectively, positive Tajima’s *D* and total nucleotide diversity were observed at or around codon site 200 in the sliding window analyses (Fig. 2), and these peaks are a typical signature of balancing selection (cf. [13, 14]). No marked peak or plateau for polymorphism or for Tajima’s *D* was observed for the second putative selected site (site 173) in the present study (Fig. 2), in agreement with the previous study [34] which showed a weaker signal of balancing selection for site 173 than for site 200.

## Conclusions

The *PgiC1* gene in *F. ovina* represents one of the few reported cases in which the levels of nucleotide polymorphism differ substantially between the 5’ and 3’ portions of a gene. The present study suggests that the contrasting levels of nucleotide polymorphism between the two portions of *PgiC1* may have resulted from different selective regimes in the two gene portions. Relatively strong purifying selection appears to have reduced the level of polymorphism within the 3’ portion, whereas balancing selection may have contributed to the maintenance of the polymorphism in the 5’ portion of the sequence. A high overall level of recombination and a low level of LD within *PgiC1* may have allowed partially independent selection and evolution within the two portions of the gene.

## Additional files

Additional file 1: **File S1.** The aligned multiple-species homologous amino acid sequences acquired from the Consurf Server. (TXT 96 kb)

Additional file 2: Table S1. The total nucleotide diversity (*π*_T_) for each studied *PgiC1* exon. (DOCX 47 kb)

Additional file 3: Table S2. Estimates of *ω* and *ρ* (made with omegaMap) for each analyzed *PgiC1* codon. (XLS 119 kb)

Additional file 4: Table S3. Comparison (Hudson-Kreitman-Aguadé test) between the 5’ and 3’ portions of the sequenced *F. ovina PgiC1* in terms of level of polymorphism and level of divergence from the outgroup *F. altissima*. (DOC 33 kb)

Additional file 5: Figure S1. Results for the sliding window analyses of, respectively, Tajima’s *D*, Fay and Wu’s *H*, and *K*a/*K*s. The ticks on the x axis represent the boundary of each analysed *PgiC1* exon within the *PgiC1* coding sequence. In *F. ovina*, *PgiC1* exons 5–12 encode the small domain of a PGI monomer while exons 13–21 encode the large domain. The three stars on the x axis represent the three active site residues (equivalent to Lys516, Glu360, and His391 in *F. ovina*) that are directly involved in the PGI isomerization reaction [35]. The dotted vertical line highlights a position where both *D* and *H* have marked valleys. (TIF 1634 kb)

Additional file 6: Table S4. The estimated (normalized) conservation scores for each PGI amino acid site. (XLS 49 kb)

## Abbreviations

HKA: Hudson-Kreitman-Aguadé test
HPD: Highest posterior density
LD: Linkage disequilibrium
MHC: The major histocompatibility complex
MK test: MacDonald and Kreitman’s test
PGI: Phosphoglucose isomerase
SNP: Single nucleotide polymorphism
