# Supplementary material for: Contrasting patterns of nucleotide polymorphism suggest different selective regimes within different parts of the PgiC1 gene in Festuca ovina L
Source: Hereditas. 2017 May 18;154:11. doi: 10.1186/s41065-017-0032-6 (PMC5437402; doi:10.1186/s41065-017-0032-6)
Supplement: Supplementary file 2 — The total nucleotide diversity (π T) for each studied PgiC1 exon. (DOCX 47 kb) [file 41065_2017_32_MOESM2_ESM.docx]

Table S1. The total nucleotide diversity (*π*_T_) for each studied *PgiC1* exon.

| *PgiC1* 5’ portion | | *PgiC1* 3’ portion | |
| --- | --- | --- | --- |
| Exon no. | *π*_T_ | Exon no. | *π*_T_ |
| 1 | 0.00000 | 13 | 0.00588 |
| 2 | 0.02130 | 14 | 0.00233 |
| 3 | 0.03226 | 15 | 0.00000 |
| 4 | 0.01314 | 16 | 0.00516 |
| 5 | 0.02343 | 17 | 0.00216 |
| 6 | 0.01986 | 18 | 0.00513 |
| 7 | 0.00882 | 19 | 0.00128 |
| 8 | 0.02427 | 20 | 0.00193 |
| 9 | 0.03356 | 21 | 0.00296 |
| 10 | 0.00000 | 22 | 0.00909 |
| 11 | 0.01485 |  |  |
| 12 | 0.00339 |  |  |
