# Supplementary material for: Contrasting patterns of nucleotide polymorphism suggest different selective regimes within different parts of the PgiC1 gene in Festuca ovina L
Source: Hereditas. 2017 May 18;154:11. doi: 10.1186/s41065-017-0032-6 (PMC5437402; doi:10.1186/s41065-017-0032-6)
Supplement: Supplementary file 4 — Comparison (Hudson-Kreitman-Aguadé test) between the 5’ and 3’ portions of the sequenced F. ovina PgiC1 in terms of level of polymorphism and level of divergence from the outgroup F. altissima. (DOC 33 kb) [file 41065_2017_32_MOESM4_ESM.doc]

**Table S3. Comparison (Hudson-Kreitman-Aguadé test) between the 5’ and 3’ portions of the sequenced *F*. *ovina* *PgiC1* in terms of level of polymorphism and level of divergence from the outgroup *F*. *altissima*.**

|  | 5’ portion | 3’ portion |
| --- | --- | --- |
| Polymorphism within *F*. *ovina* |  |  |
| Number of segregating sites (observed) | 40 | 14 |
| Number of segregating sites (expected^a^) | 31.47 | 22.53 |
| Divergence between *F*. *ovina* and *F*. *altissima* |  |  |
| The average number of pairwise differences (observed) | 29.62 | 35.83 |
| The average number of pairwise differences (expected^a^) | 38.15 | 27.30 |
| Total number of synonymous sites considered | 570 | 612 |
| Number of sequences analyzed | 29 | 29 |

**NOTES.** Because parts of the coding sequence are not available for the outgroup sequence from *F*. *altissima*, the 5’ and 3’ portions of *F*. *ovina* *PgiC1* that were considered in the test span, respectively, coding sequence nucleotide positions 259-828 & 919-1530.

^a^ neutral expectation
