# Supplementary figures and images for: Contrasting patterns of nucleotide polymorphism suggest different selective regimes within different parts of the PgiC1 gene in Festuca ovina L
Source: Hereditas. 2017 May 18;154:11. doi: 10.1186/s41065-017-0032-6 (PMC5437402; doi:10.1186/s41065-017-0032-6)

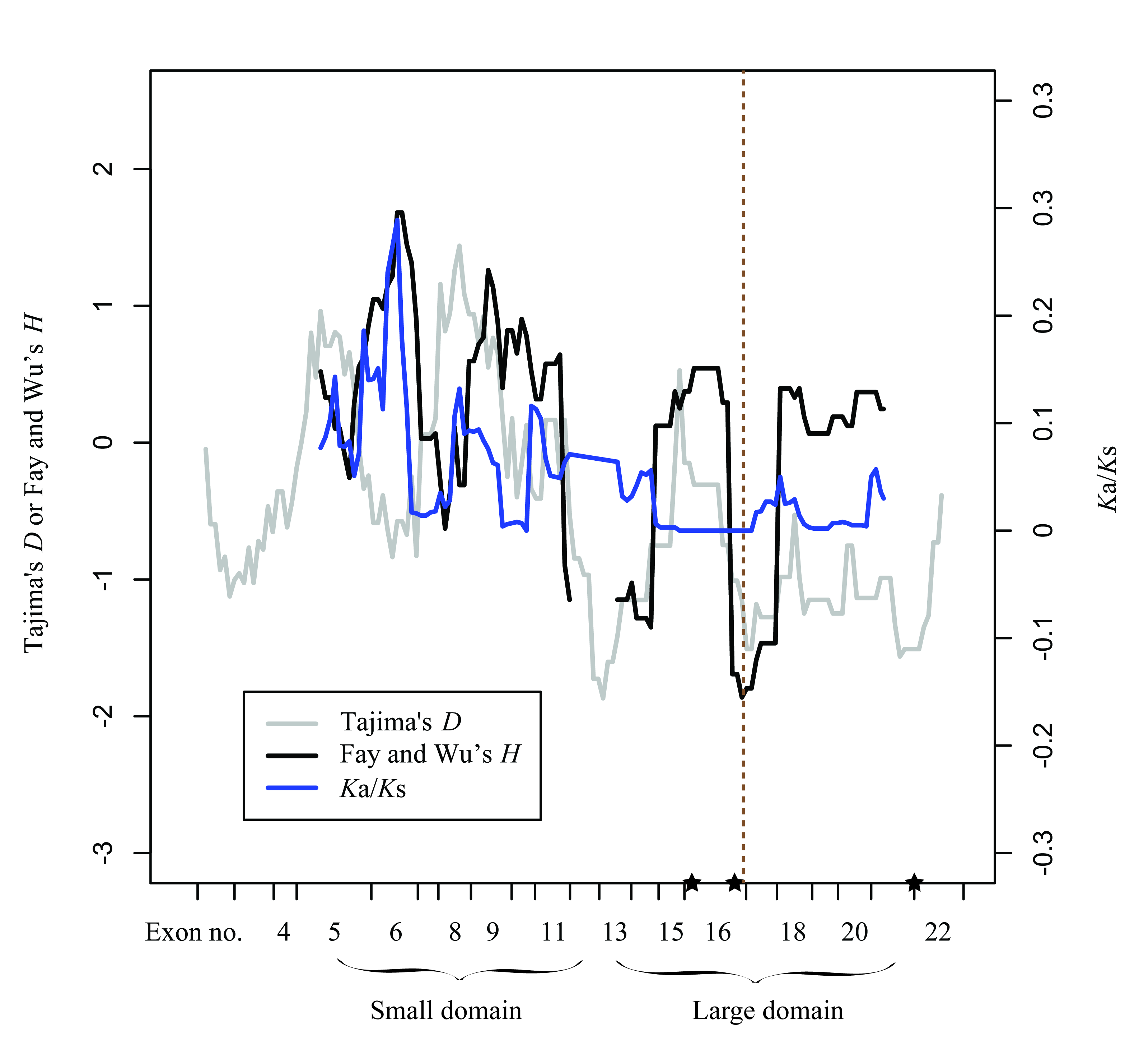

Supplement: Supplementary file 5 — Results for the sliding window analyses of, respectively, Tajima’s D, Fay and Wu’s H, and Ka/Ks. The ticks on the x axis represent the boundary of each analysed PgiC1 exon within the PgiC1 coding sequence. In F. ovina, PgiC1 exons 5–12 encode the small domain of a PGI monomer while exons 13–21 encode the large domain. The three stars on the x axis represent the three active site residues (equivalent to Lys516, Glu360, and His391 in F. ovina) that are directly involved in the PGI isomerization reaction [35]. The dotted vertical line highlights a position where both D and H have marked valleys. (TIF 1634 kb) [file 41065_2017_32_MOESM5_ESM.tif]
